# Coffee in Poisoning by Acetate of Morphia

**Published:** 1847-07

**Authors:** 


*Coffee in Poisoning by Acetate of Morphia.*—A patient swallowed atone dose *ten grains* and three quarters of acetate of morphia.—Tartar emetic was immediaely given, but without producing vomiting. About three hours after the accident, and while the patient was in a state of deep coma, a highly concentrated solution of coffee with the solid residue was given to him. The paient swallowed about ten ounces in twelve hours. The coma disappeared, and he perfectly recovered.—*Gaz. Medicale.*

